# ALX/FPR2 Receptor Activation by Inflammatory (fMLFII) and Pro-resolving (LXA_4_ and RvD3) Agonists

**DOI:** 10.1021/acsphyschemau.5c00008

**Published:** 2025-06-06

**Authors:** Vinicius S. Nunes, Charles N. Serhan, Odonírio Abrahão, Alexandre P. Rogério

**Affiliations:** † Programa de Pós-Graduação em Produtos Bioativos e Biociências, Universidade Federal do Rio de Janeiro, Avenida Aluizio da Silva Gomes 50, Macaé 27930-560, Brasil; ‡ Center for Experimental Therapeutics and Reperfusion Injury, Department of Anesthesiology, Perioperative and Pain Medicine, MassGeneral Brigham (MGB) and Harvard Medical School, 60 Fenwood Road, Boston, Massachusetts MA 02115, United States; § Laboratório de Química Computacional Medicinal, 74348 Universidade Federal do Triângulo Mineiro, Praça Manuel Terra 330, Uberaba 38025-050, Brasil; ∥ Laboratório de Imunofarmacologia Experimental, Universidade Federal do Triângulo Mineiro, Rua Vigário Carlos 100, Uberaba 38025-350, Brasil

**Keywords:** GPCR, ALX/FPR2 receptor, lipoxin A4, resolvin RvD3, molecular dynamics, pro-resolving mediators, inflammation

## Abstract

Nine structures of the ALX/FPR2 receptor are currently deposited in the PDB. In seven structures, the receptor is complexed with formylated peptides. In all seven structures, residue D106 is indicated as acting in the ALX/FPR2 receptor activation in addition to residues R201 and R205. Here, we performed docking simulations and long-term molecular dynamics simulations to investigate the ALX/FPR2 receptor activation using two pro-resolution agonists (lipoxin A4 (LXA_4_) and resolvin D3 (RvD3)) and a formylated peptide pro-inflammatory agonist (fMLFII). We have analyzed the receptor’s activation state, electrostatic interactions, and the binding affinities of the complexes receptor-agonist using the MM/PBSA approach. The results showed that LXA_4_ and fMLFII kept the receptor in an active state by a higher simulation time when compared to RvD3. Only R201 and R205 were considered key residues in the ALX/FPR2 receptor activation by all agonists. The electrostatic interaction analysis confirmed the importance of these residues in ALX/FPR2 receptor activation. Furthermore, only fMLLII showed interactions with residue D106. The binding free energy calculations indicated that the electrostatic component significantly binds the agonists to the receptor. Overall, the results from this study provide new insights into the ALX/FPR2 receptor activation mechanisms, reinforcing the role of critical residues and interactions in the binding of pro-resolution and inflammatory agonists.

## Graphical abstract

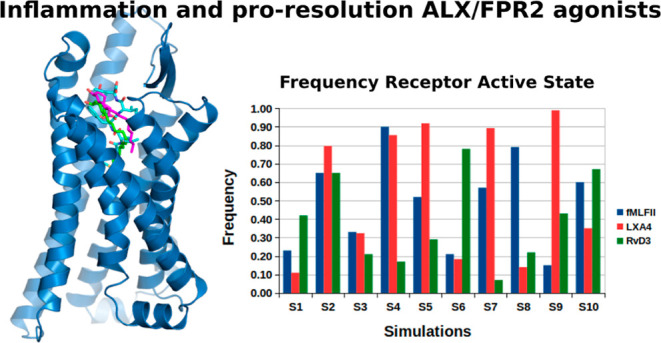

## Introduction

The formyl peptide receptors (FPRs) are a group of G protein-coupled chemoattractant receptors that play essential roles in host defense and inflammation. There are three gene codes for humans: FPR1, FPR2, and FPR3.^1,2^ FPR2 recognizes diverse formyl peptides (agonists derived from bacteria) and nonformylated peptides, for example, derived from viruses.^2,3^ In addition, FPR2 has been shown to be a receptor for bioactive eicosanoid lipid molecules known as the specialized pro-resolving lipid mediators (SPMs) such as lipoxin A_4_ (LXA_4_) and resolvins D1 (RvD1) and D3 (RvD3).^4,5^ FPR2 is often termed ALX/FPR2 since it binds to LXA_4_. While formyl peptides act on ALX/FPR2 to induce chemotaxis of immune cells and initiate numerous inflammatory processes,^6^ SPMs induce the resolution of inflammations.^7^ SPMs accelerate the reduction of resolution intervals,^8,9^ demonstrating anti-inflammatory effects but without immunosuppressive effects, enhancing macrophage phagocytosis.^7,10^

In 2020, Schmitz Nunes and colleagues proposed a model for the ALX/FPR2 receptor 3D structure complexed with the fMLFK peptide, a pro-inflammatory agonist, and the AT-RvD1, an epimer of RvD1, a pro-resolution agonist, and a mechanism of its activation based on molecular dynamics simulations.^11^ The authors showed that the two central residues (R201 and R205) act in ALX/FPR2 receptor activation in both agonists. In addition, it was demonstrated that the electrostatic interactions trigger the receptor’s activation. The development of cryo-electron microscopy (Cryo-EM) allowed nine ALX/FPR2 receptor structures which were deposited in the Protein Data Bank (PDB) in the last three years.^2,3,12–14^ Seven of them were complexed with formylated peptides. In all seven structures, residue D106 is indicated as acting in the ALX/FPR2 receptor activation, in addition to residues R201 and R205. On the other hand, Nunes and colleagues (2023) recently performed molecular docking simulations of RvD1 and AT-RvD1 with the 6OMM structure of the ALX/FPR2 receptor and showed that both RvD1 and AT-RvD1 activated the ALX/FPR2 receptor via R201 and R205, without the participation of D106.^15^ In 2013 Cooray et al. demonstrated that LXA_4_ stimulates ALX/FPR2 dimers to produce IL-10, an anti-inflammatory cytokine, as a novel ligand-bias mechanism.^16^ Several studies have shown that some agonists can induce or stabilize the GPCR receptors’ dimeric form.^17–21^

Here, using the ALX/FPR2 receptor’s structure 7T6V complexed with fMLFII peptide, we performed docking simulations of LXA4 and RvD3 in the receptor. Next, we performed long-term molecular dynamics simulations with the FPR2@fMLFII, FPR2@LXA4, and FPR2@RvD3 complexes. Through these simulations, we studied the activation mechanisms of the ALX/FPR2 receptor involving these proinflammatory (fMLFII) and proresolution (LXA4 and RvD3) agonists. Along the same lines, we investigated which residues were essential in the interaction between the ALX/FPR2 receptor and agonist and in its activation. We also analyzed the binding affinities of these three complexes using the Molecular Mechanics Poisson–Boltzmann Surface Area approach (MM/PBSA).

## Materials and Methods

### Protein and Resolvins Were Used in the Simulations

From PDB 7T6V structure,^3^ downloaded OPM (Orientations of Proteins in Membranes) database,^22^ we extracted the complex FPR2@fMLFII. The ALX/FPR2 receptor is in the active state. The LXA_4_ (CID5280914) and RvD3 (CID71665428) structures were downloaded from the PubChem database.^23^ Protein and ligands were prepared by adding hydrogens at pH 7.3 using Maestro Academic License version 2018–3.^24^ For parametrization of LXA_4_ and RvD3 structures, we used the CHARMM36 general force field (CGEnFF) toolkit (https://cgenff.umaryland.edu/).^25,26^ The fFMLFII peptide was parametrized using the CHARMM36 protein force field.

### LXA_4_ and RvD3 Molecular Docking Simulations

To prepare the ALX/FPR2 receptor and, LXA_4_ and RvD3 structures, we used the MGLTools.^27^ Molecular docking simulations were performed using the AutoDock Vina.^28^ For the docking simulations, we used the following box settings: (i) size box, *x* = 30 Å, *y* = 30 Å, and *z* = 30 Å; center coordination, *x* = 0, *y* = 0, and *z* = 11.

### Systems’ Preparation

For molecular dynamics simulations, we prepared three systems in the CHARMM-GUI.^29,30^ For each system, we used a heterogeneous membrane with the following composition: 76% POPC (57 molecules) and 24% cholesterol (18 molecules) for each leaflet. The each system’s volume was 73 Å × 73 Å × 97 Å. We used the TIP3P water model (14 Å water thickness), and the systems were ionized with 0.15 M of NaCl. The total number of atoms in the FPR2@fMLFII system was 52,788, in the FPR2@LXA_4_ system was 51,822, and in the FPR2@RvD3 system was 51,825.

### Molecular Dynamic Simulations (MD)

After assembling the three systems, we performed an optimization using conjugate gradient method for 10,000 steps and the steepest descent during 5000 steps. Next, systems equilibration were performed in four stages: (i) heating the system from 100 to 150 K for 125 ps under the *NVT* ensemble with weak position restraints for the protein and lipids heavy atoms, using a weight of 2 kcal/molÅ^2^; (ii) heating from 150 to 250 K for 125 ps under the *NVT* ensemble with weak position restraints for the protein and lipids heavy atoms, using a weight of 2 kcal/mol Å^2^; (iii) heating from 250 to 310 K for 250 ps under the *NVT* ensemble with weak position restraints for the protein and lipids heavy atoms, using a weight of 2 kcal/mol Å^2^; (iv) MD under the *NPT* ensemble at a constant temperature of 310 K for 1000 ps with position restraints for the protein and lipids heavy atoms, using a weight of 2 kcal/mol Å^2^; (v) a new MD under the *NPT* ensemble at a constant temperature of 310 K for 1000 ps with position restraints for only the protein heavy atoms, using a weight of 1 kcal/mol Å^2^; and finally, (vi) a MD under the *NPT* ensemble at a constant temperature of 310 K for 5000 ps with no position restraints for any atoms. After the equilibration step, we performed the production step for 2.4 μs in the *NPT* ensemble using an integration step of 1 fs. For temperature control, we used the Langevin thermostat.^31^ We used the Monte Carlo barostat with semi-isotropic coupling and constant surface tension on the XY plane for pressure control.^32^ We also used the SHAKE algorithm to constrain the all bonds involving hydrogen.^33^ For short-range electrostatic interactions and van der Waals interactions, we applied a cutoff of 11 Å. The algorithm for the electrostatic interaction integration applied was the PME.^34^ The force field used in the simulations was CHARMM36.^35,36^ During production, the receptor poses were saved every 80 ps. Ten simulations (replicas) of 2.4 μs for each system, changing the atom’s initial velocity seed, were performed. These ten simulations totaled 24 μs of simulation. All molecular dynamics simulations were performed with AMBER2018 ^37^ on the SDumont supercomputer (Laboratório Nacional de Computação Científica—Brazil) using Nvidia Tesla V100 GPUs.

### Molecular Dynamic Simulation Analysis

For electrostatic interactions (hydrogen bond and salt bridge), we used CPPTRAJ2018 ^37^ and VMD^38^ tools, respectively. For the TMH3-TMH6 distance, we used the average distance (*U̅*_S_) of about I116–V247 (*U*_1_) and R123-L243 (*U*_2_) distances in the 7T6V structure (*U̅*_S_ = 10.8Å). The same average distance in each frame is *U̅*_F_.

### MM/PBSA Energy Analyses

From each simulation, we used 1000 frames collected along the entire trajectory at intervals of 30 frames. The calculations were performed using the MMPBSA.py script,^39^ the AMBER2018 package, and the MM/PBSA (Molecular Mechanics Poisson–Boltzmann Surface Area) method.^40,41^ For files preparations, we used the protocol shown in the Amber tutorial (https://ambermd.org/tutorials/advanced/tutorial3/py_script/index.htm). The parameters relating to the membrane dielectric constant (*enem*) and the protein dielectric constant (*indi*) of the MMPBSA.py program were adjusted to carry out the calculations.

In the MM/PBSA method, the binding free energy is calculated by eq 1

1 $$\Delta G_{bind}=\Delta G_{complex}-\Delta G_{ligand}-\Delta G_{protein}$$

where ΔG_complex_, Δ*G*_ligand_, and Δ*G*_protein_ are the free energy of the protein–ligand complex, the ligand, and the protein, respectively. Each term of eq 1 is written as follows

2 $$\Delta G_{MM/PBSA}=\Delta E_{MM}+\Delta G_{PolarSolv}+\Delta G_{ApolarSolv}$$

where Δ*E*_MM_ is the sum of electrostatic and nonelectrostatic potentials of the molecular mechanics (eq 3).^40,41^

3 $$\Delta E_{MM}=\Delta E_{elect}+\Delta E_{nelect}$$

The apolar solvation (Δ*G*_ApolarSolv_) free energy is modeled as two terms: the cavity term and the dispersion term. The cavity term was calculated by eq 4

4 $$\Delta G_{ApolarSolv}=\int_{0}^{1}{\left\langle \frac{dH\lambda }{d\lambda }\right\rangle }_{\lambda }d\lambda$$

where *H* is a parametrized Hamiltonian, and λ is the coupling coefficient.^42^ The cavity term is still computed as a term linearly proportional to the molecular solvent.^37,40,41^ For SASA calculation, AMBER2018 uses the LCPO algorithm.^37,43^

In the PBSA approach, polar solvation contribution, including the electrostatic field and the solvent reaction field, may be computed by solving the PB eq 5

5 $$\nabla \times \varepsilon \left(r\right)\nabla \phi \left(r\right)=-4\pi \rho \left(r\right)-4\pi \lambda \left(r\right)\sum_{i}z_{i}c_{i}\exp\left(-z_{i}\phi \left(r\right)/k_{B}T\right)$$

where ε­(*r*) is the dielectric constant, φ­(*r*) is the electrostatic potential, ρ­(*r*) is the solute charge, λ­(*r*) is the Stern layer masking function, *z*_*i*_ is the charge of ion type *i*, *c*_*i*_ is the bulk number density of ion type *i* far from the solute, *k*_B_ is the Boltzmann constant, and *T* is the temperature; the summation is over all different ion types.^37,40,41^

## Results

As previously described, the fMLFII peptide is found in the 7T6V structure, and we used the peptide position on the receptor to adjust the box position in LXA_4_ and RvD3 docking simulations. Figure 1 shows the position of the fMLFII peptide within the ALX/FPR2 receptor (Figure 1A) and the superposition of LXA_4_, RvD3, and the peptide in the ALX/FPR2 receptor-binding site (Figure 1B). fMLFII is a pro-inflammatory agonist, and its FPR2 receptor binding and activation site is the ALX/FPR2 receptor core (Figure 1A). LXA_4_ and RvD3 are pro-resolution agonists of inflammation.^4,44,45^ Our previous work has shown that the core of the receptor is also the site of binding and activation of the ALX/FPR2 receptor by the pro-resolution agonists RvD1 and AT-RvD1.^11,15^

**Figure 1:**
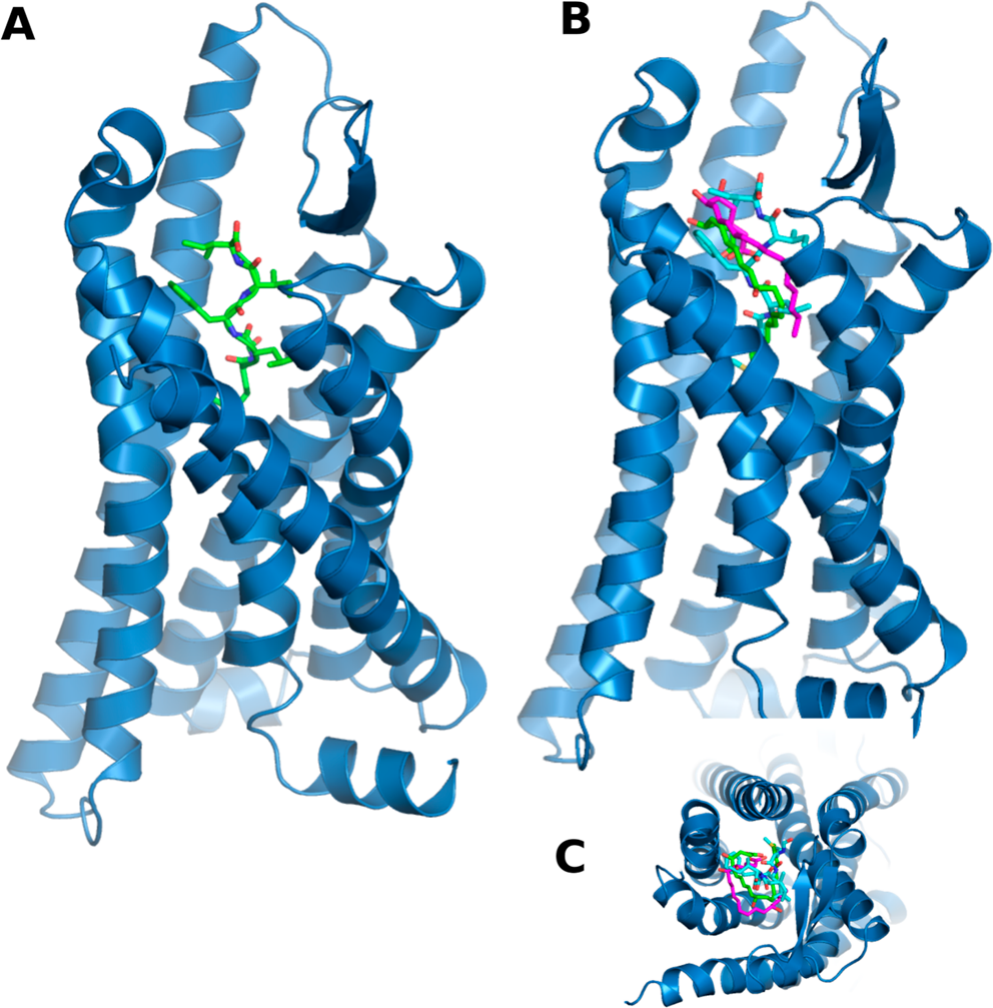
(A) 7T6V ALX/FPR2 receptor structure (blue), fMLFII (green), LXA_4_ (cyan), and RvD3 (purple). (B) Superpose fMLFII and molecular docking poses of LXA_4_ (B.A. 7.3 kcal/mol) and RvD3 (B.A. 6.7 kcal/mol). (C) Molecular docking upper view. B.A. = Vina’s binding affinity.

We started the MD simulations after the LXA_4_ and RvD3 docking simulations. Three systems involving the ALX/FPR2 receptor, agonists, membrane, and explicit solvent were prepared and equilibrated (Figure S1): FPR2@fMLFII, FPR2@LXA4_4_ and FPR2@RvD3. For the three systems, we checked the time that the receptor remained in the active state, the electrostatic interactions involving the receptor and agonists, and the binding free energy (Δ*G*_Bind_) for each agonist.

The first analysis of the MD simulations was to verify the receptor’s permanence in the active state, where *U̅*_S_ is the average distance of about I116–V247 (*U*_1_) and R123–L243 (*U*_2_) distances in the 7T6V structure (*U̅*_S_ = 10.8 Å), and *U̅*_F_ is the same average distance in each frame of trajectories. In this analysis (Figure 2), as a threshold for the receptor’s active state, we use the average distance between pairs I116–V24 and R123–L243 (Figure S5). We analyzed the frame’s frequency where *U̅*_F_ ≥ *U̅*_S_ (Figure 2A and Table S1) and the variation of *U̅*_F_ over the 24 μs of the simulation time of each complex: FPR2@fMLFII (Figures 2B and S1), FPR2@LXA4 (Figures 2C and S1), and FPR2@RvD3 (Figures 2D and S1). Our results showed that in the FPR2@fMLFII simulations, *U̅*_F_ ≥ *U̅*_S_ was 50%. In the FPR2@LXA_4_ simulations, *U̅*_F_ ≥ *U̅*_S_ was 56%, while in the FPR2@RvD3 simulations, *U̅*_F_ ≥ *U̅*_S_ was 39%. LXA_4_ was the agonist that maintained the receptor’s active state more frequently among the three agonists. It is interesting to highlight that in previous work by our group, we showed that RvD1 and AT-RvD1 kept the receptor in the active state in at least 70% of the analyzed frames.^15^

**Figure 2:**
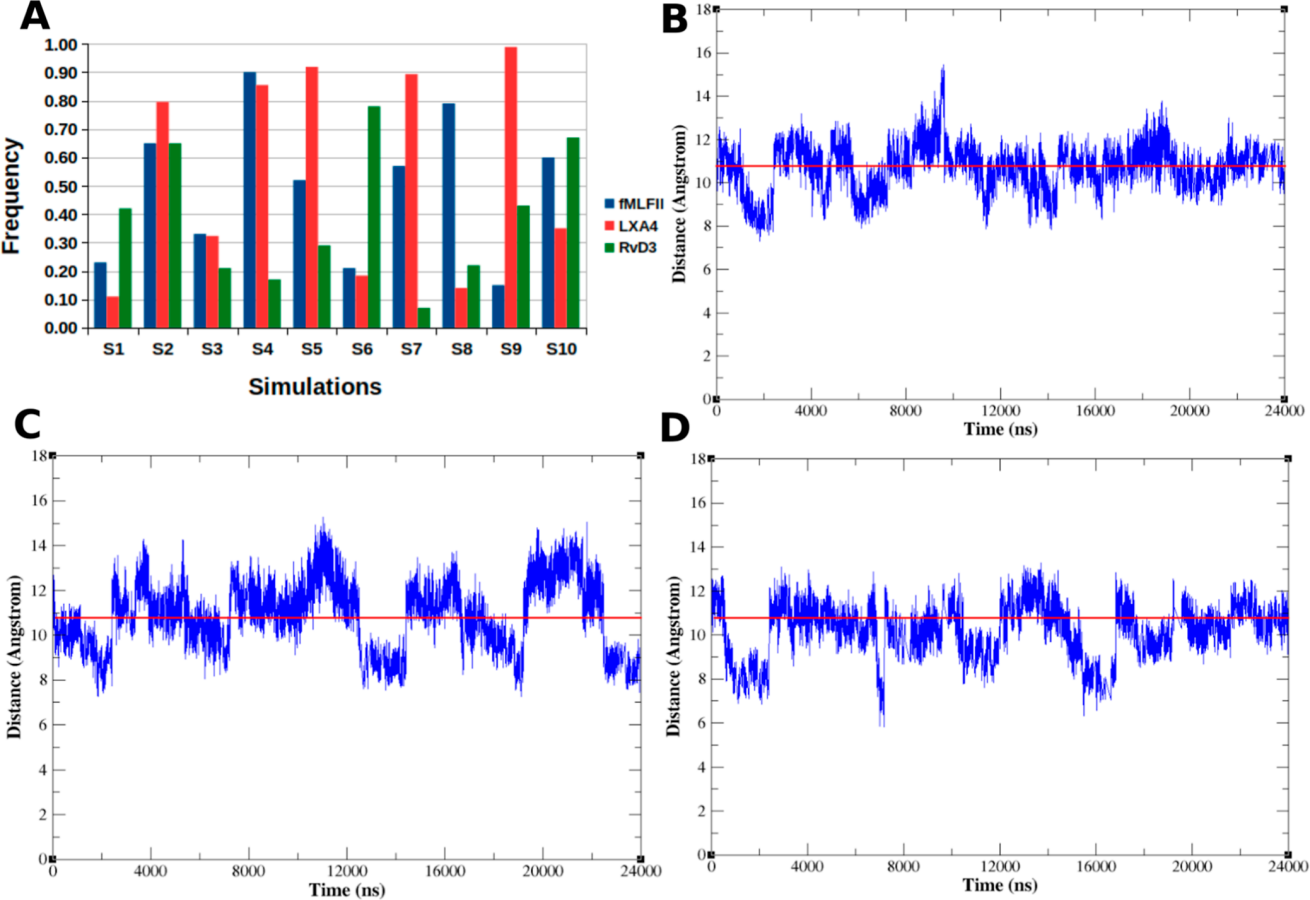
(A) Frames’ frequency where *U̅*_F_ ≥ *U̅*_S_. *U̅*_F_ variation on (B) FPR2@fMLFII simulations, (C) FPR2@LXA_4_ simulations, and (D) FPR2@RvD3 simulations. Red line is the 10.8Å threshold. (B–D) Simulation time merging of the ten replicates.

After verifying the frequency of the ALX/FPR2 receptor’s active state in the molecular dynamic simulations, we analyzed the event of intermolecular interactions such as hydrogen bonds and salt bridges (Figure 3A; Tables S2–S4). Figure 3 shows that the two main fMLFII electrostatic interactions are D106, R201, and R205 (Figure 3A; Table S2), and this interaction occurs mainly via the N-formyl group of formylated methionine (Figure 3B). In the case of D106, the interaction occurs via the amine of the N-formyl group and for residues R201 and R205 via the aldehyde of the N-formyl group (Figure 3B). For LXA4, the main interactions were two salt bridges between the carboxylate group and residues R201 and R205 (Figure 3C,D; Table S3). The same could be observed for RvD3 (Figure 3E,F; Table S4). Both residues are confirmed by the ALX/FPR2 receptor structures deposited in the PDB, and only the structures with formylated peptides show the D106 interaction.^2,3,12,14^ These results are in agreement with previous results using other SPMs (RvD1 and AT-RvD1) Such results indicated that residues R201 and R205 were pivotal in the ALX/FPR2 receptor activation for all agonists.

**Figure 3:**
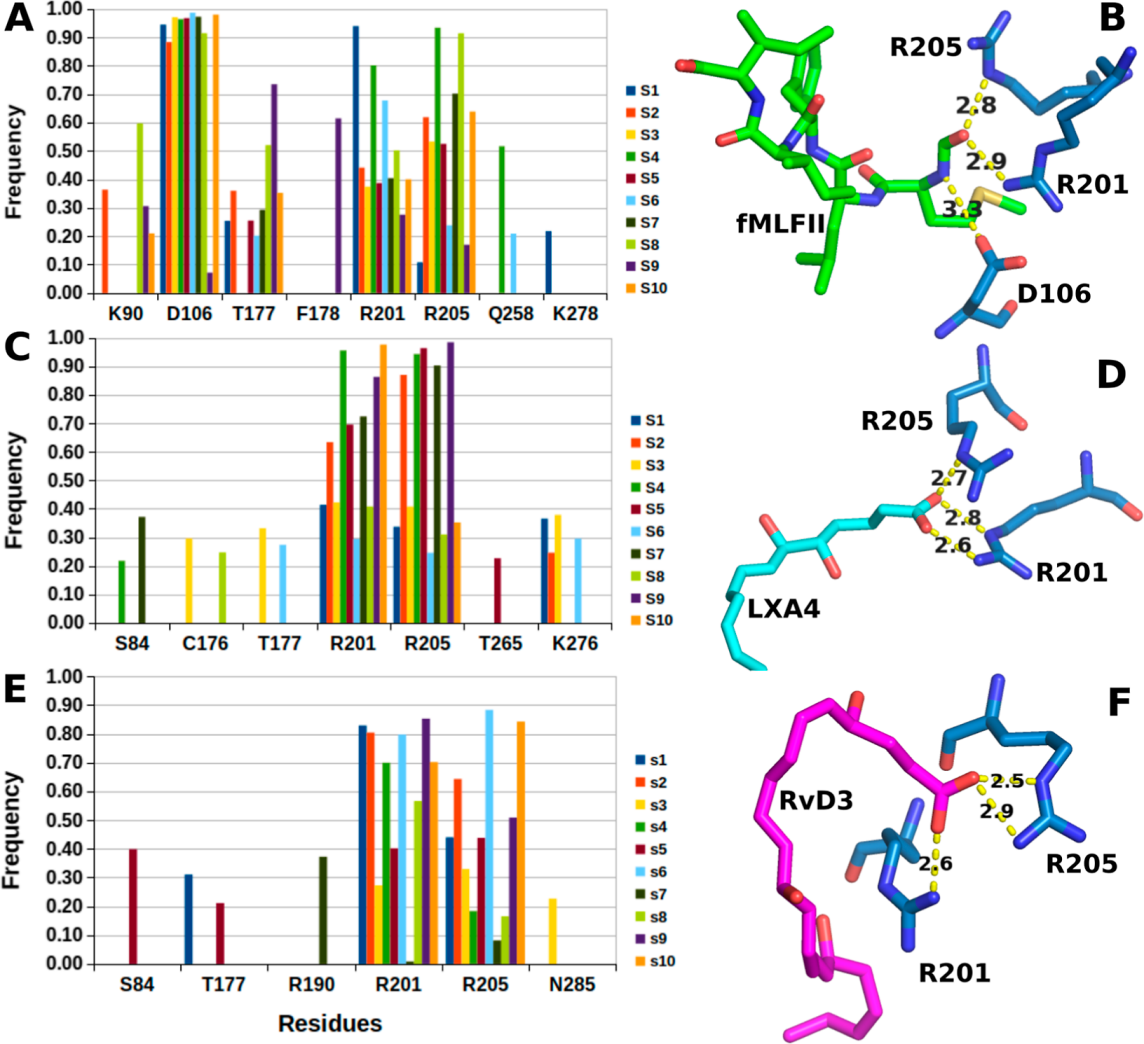
(A) Electrostatic interaction frequency in FPR2@fMLFII simulations, (C) FPR2@LXA4 simulations, and (E) FPR2@RvD3 simulations. Poses that show electrostatic interactions in (B) FPR2@fMLFII simulations, (D) FPR2@LXA4 simulations, and (F) FPR2@RvD3 simulations.

After analyzing the electrostatic interaction frequency, we performed binding free energy (Δ*G*_Bind_) calculations for the three complexes simulated. Figure 4 shows the Δ*G*_Bind_ variation (Figure 4A; Table S5) on all simulations and Δ*G*’s contribution to the three residues with the highest electrostatics interaction frequencies (D106, R201, and R205) (Figure 4B–D; Table S7).

**Figure 4:**
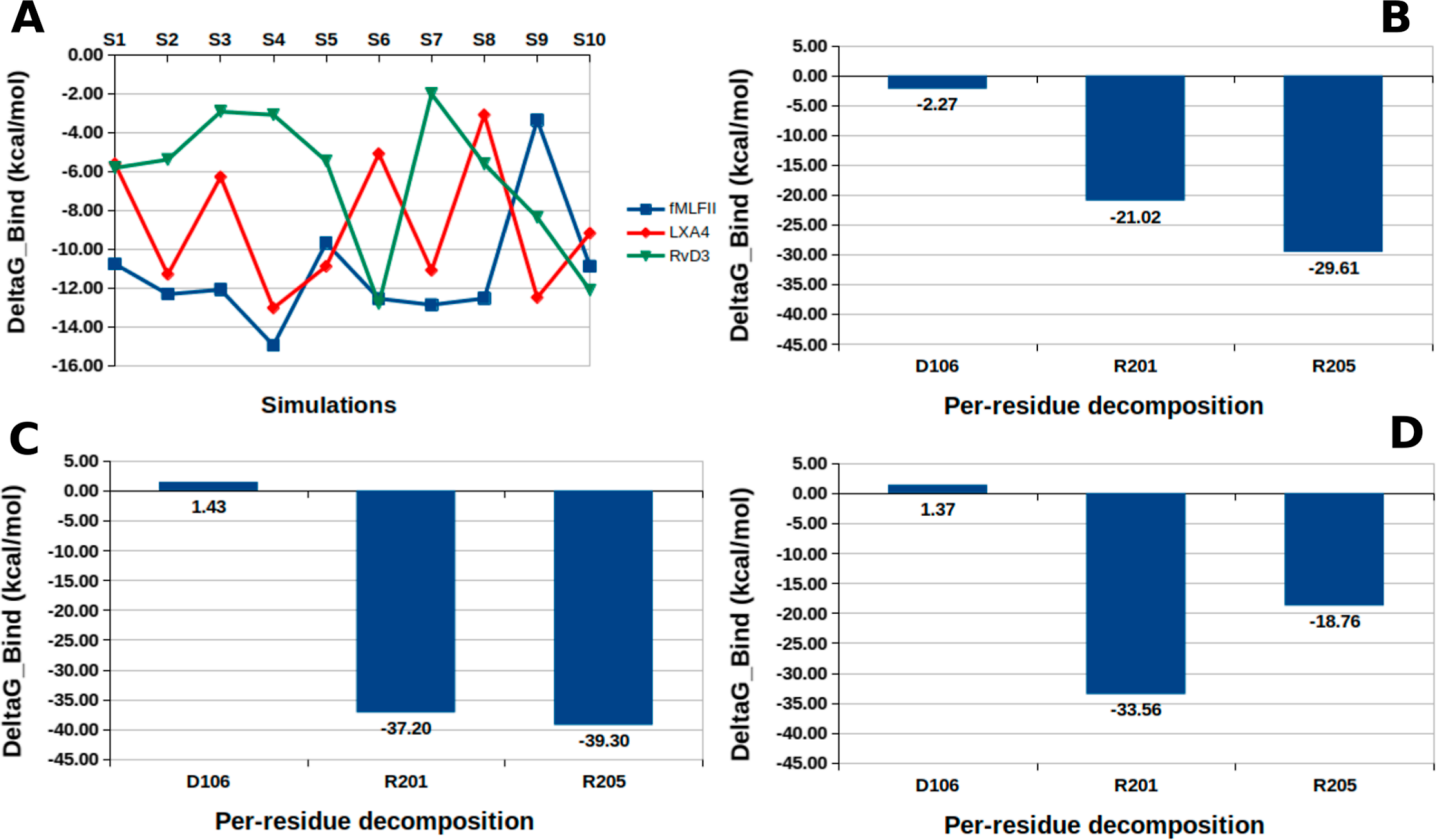
(A) Δ*G*_Bind_ averages for all simulations (see Table S6). Δ*G*’s contribution to the three residues with the highest electrostatics interaction frequencies (D106, R201, and R205): for the FPR2@fMLFII simulations (B), FPR2@LXA_4_ simulations (C), and FPR2@RvD3 simulations (D).

Regarding the ΔGBind variation (Figure 4A; Table S5), our results showed that the FPR2@fMLFII complex has an average Δ*G*_Bind_ of −11.04 kcal/mol, while the standard deviation is 3.16 kcal/mol (Table S5). For the FPR2@LXA_4_ complex, the average ΔG_Bind_ was −8.87 kcal/mol and the standard deviation was 3.55 kcal/mol (Table S5). Finally, for the FPR2@RvD3 complex, the average Δ*G*_Bind_ was −6.36 kcal/mol and the standard deviation was 3.34 kcal/mol (Table S5). It is necessary to highlight that the binding Δ*G*_Bind_ values calculated by the MMPBSA method for the three agonists were close to the experimental deltaG obtained from EC_50_ (see Table S8).

Regarding the contribution of residues, the three hotspot residues for fMLFII were indicated in the electrostatic interaction analysis, D106, R201, and R205 (Figure 4B–D; Table S7). For the FPR2@LXA_4_ and FPR2@RvD3 complexes, the two hotspots were R201 and R205. Such results were confirmed by the electrostatic interaction analysis of other studies,^11,15^ and mutagenesis studies.^46,47^ In other words, R201 and R205 are two pivotal residues in ALX/FPR2 receptor activation. Our results suggest that D106 was not critical in receptor activation. In previous studies by our group, we showed that D106 was not fundamental in the ALX/FPR2 receptor activation by RvD1 and AT-RvD1 and fMLFK.^11,15^

## Discussion

The formyl peptide receptors are a group of G protein-coupled chemoattractant receptors that play important roles in host defense and inflammation.^1^ Three genes coding for human FPRs were cloned, including FPR1, FPR2, and FPR3.^1,2^ FPRs, together with complement C5a peptide, leukotriene B4, prostaglandin D2, and chemokines receptors, constitute a group of Gi-coupled chemoattractant receptors that belong to the rhodopsin-like Class A GPCR.^47^ The ALX/FPR2 can recognize diverse formyl peptide agonists derived from various bacteria and nonformylated peptides from viruses.^2,6^ In addition, ALX/FPR2 is the receptor of some SPMs.^9^ ALX/FPR2 is implicated in the pathogenesis of chronic inflammatory diseases, including asthma,^48^ Alzheimer’s disease,^14,49^ and cardiovascular diseases.^1^ In particular, ALX/FPR2 agonists that can specifically activate the resolution pathways represent a new therapeutic frontier.^50,51^ Fiore and collaborators made the first description of the ALX/FPR2 receptor, but its 3D structure remained unresolved.^52,53^ Since 2020, many structures of the ALX/FPR2-Gi complex were published. However, receptor activation remains unclear, for both inflammatory agonists and pro-resolution agonists.

Our results showed that the ALX/FPR2 receptor remained in the active state for a longer time with pro-resolution agonist LXA_4_ and that the Δ*G*_Bind_ contribution of residue R205 was the lowest among the three agonists. Regarding the contribution of residue R201, the lowest energy was observed in the FPR2@LXA_4_ and FPR2@RvD3 simulations. In 1997, Miettinen and colleagues demonstrated that the mutagenesis of ALX/FPR2 residues R201 and R205 disabled the receptor from activating G protein by formylated peptides and suggested that both residues would be essential for its activation.^46^ In another study, Wang and colleagues demonstrated a mutagenesis in the prostaglandin D2 receptor at the K210 (same position of R205 in the ALX/FPR2 receptor) which showed to be pivotal for receptor activation.^47^ In 2020, using molecular dynamics simulations, Schmitz Nunes and colleagues showed that residues R201 and R205 act directly in the ALX/FPR2 activation by fMLFK (a formylated peptide, pro-inflammatory agonist) and AT-RvD1 (a SPM, pro-resolution agonist).^11^ Also in 2020, two structures of the FPR2 receptor complexed with synthetic peptides provided with modified methionine were published. Both studies suggested that residues R201 and R205 are fundamental to ALX/FPR2 receptor activation.^2,12^ In 2021, Nunes and colleagues, using long-term molecular dynamics simulations, showed that residues R201 and R205 were essential for the activation of the ALX/FPR2 receptor by RvD1 and its aspirin-triggered 17R epimer.^15^ In 2022, seven Cryo-EM ALX/FPR2 receptor structures were presented in two publications.^3,14^ Of the seven structures, five were complexed with formylated or synthetic peptides, one complexed with beta amyloid peptide, and one complexed with a synthetic agonist (compound C43). In all these structures, the participation of the R201 and R205 in the FPR2 activation was also confirmed.^3,14^ Therefore, all results above are in agreement with our results and suggest that residues R201 and R205 act in ALX/FPR2 receptor activation, regardless of the agonist.

The ALX/FPR2 structures complexed with formylated peptides,^3,14^ while the *N*-formylmethionine group interacted with the D106 residue. Interestingly, in the ALX/FPR2 structures with amyloid beta and compound C43, there were no interactions between D106 and agonists.^2,3,14^ Our results showed that only the fMLFII interacted with D106 via the amine of the N-formylmethionine group, and this interaction was of a hydrogen bond type (Figure 3B). Both LXA_4_ and RvD3 did not show interactions with D106. Our results showed that (i) only formylated peptides interact with D106 via the N-formyl’s amine group (amine group is positive) and (ii) LXA4 and RvD3 do not interact with D106 due to the carboxylate group (negative group).

Regarding the D106, our results suggest that D106 may have an allosteric effect on ALX/FPR2 activation. Here, it is necessary to highlight the work of Ye and collaborators.^54^ In this work, the authors constructed fluorescent biosensors of FPR2 based on single-molecule fluorescent resonance energy transfer (FRET) and used them to measure ligand-induced receptor conformational changes.^54^ They showed the presence of two allosteric binding sites on FPR2, each with high and low affinities.^54^

Still on D106, some studies involving molecular phylogenetic analysis of GPCR receptors, mainly from group A, showed that FPR and GPR32 receptors were a monophyletic group, and GPR32’s sequence was older than FPR receptor sequences.^55–58^ To illustrate these phylogenetic studies, we performed a sequence alignment of FPR and other SPM receptors. The section of alignment where D106 was located is shown in Figure 5. D106 was conserved only in FPR receptors. This suggests that the ancestral sequence did not have the aspartate residue at position 106.

**Figure 5:**
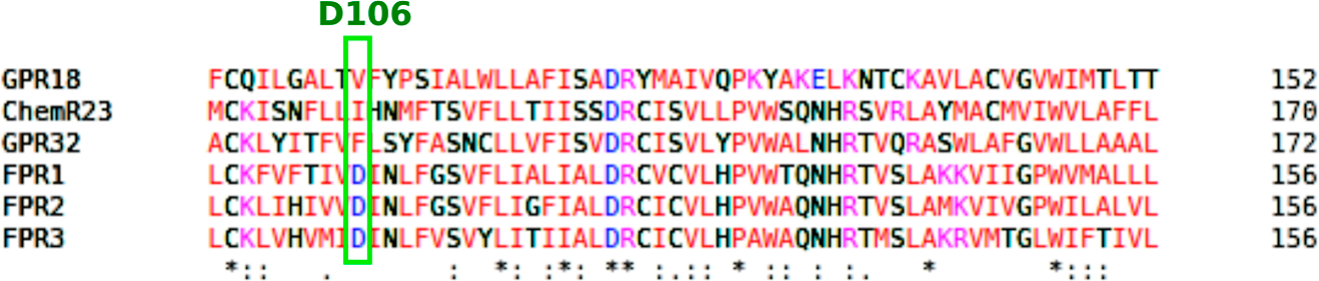
Alignment of FPR and other SPM receptor sequences. Highlighted is D106’s alignment position.

The inflammation resolution by the SPMs is very well shown in the literature.^9^ Several authors suggest the use of these molecules in the treatment of chronic inflammatory diseases.^59–61^ Furthermore, these molecules can inspire the development of new drugs. However, much still needs to be known about how SPM receptors modulate the pro-resolution response. It is crucial to know the inflammatory and pro-resolution response modulation via the ALX/FPR2 receptor for the evolution of new therapeutic approaches for chronic inflammatory diseases.

## Conclusions

Our results showed consistent evidence of ALX/FPR2 activation by inflammatory and pro-resolving agonists. In the simulations with fMLFII, the receptor remained in the active state for 50% of the total simulation time. With LXA4, this percentage was 56%, while in the simulations with RvD3, this percentage was 39%. We also observed that only fMLFII established an interaction with D106 in all simulations, suggesting that this residue has an allosteric effect on receptor activation. As for R201 and R205, our results showed that these two residues are pivotal in receptor activation regardless of the agonist. Furthermore, R201 and R205 were the residues that best contributed to the binding free energy (Δ*G*_Bind_).

## Supplementary Material



## Notes

Additional experimental details and figures and tables that complement the results obtained in the present work (PDF)

## Abbreviations

lipoxin A_4_: 5­(*S*),6­(*R*),15­(*S*)-trihydroxy-7*E*,9*E*,11*Z*,13*E*,-eicosatetraenoic acid (LXA_4_)
resolvin D3: 4*S*,11*R*,17*S*-trihydroxydocosa-5*Z*,7*E*,9*E*,13*Z*,15*E*,19*Z*-hexaenoic acid (RvD3)
fMLFII: *N*-formylmethionine-leucyl-phenylalanine-isoleucine-isoleucine
BLT1@LXA_4_: complex receptor and LXA4 agonist
BLT1@RvD3: complex receptor and RvD3 agonist
BLT1@fMLFII: complex receptor and fMLFII agonist

## References

[1] He H. Q., Ye R. D. (2017). The formyl peptide receptors: Diversity of ligands and mechanism
for recognition. Molecules.

[2] Zhuang Y., Liu H., Edward Zhou X., Kumar Verma R., de Waal P. W., Jang W., Xu T.-H. H., Wang L., Meng X., Zhao G., Kang Y., Melcher K., Fan H., Lambert N. A., Eric Xu H., Zhang C. (2020). Structure of formylpeptide receptor
2-Gi complex reveals insights into ligand recognition and signaling. Nat. Commun..

[3] Zhuang Y., Wang L., Guo J., Sun D., Wang Y., Liu W., Xu H. E., Zhang C. (2022). Molecular
recognition of formylpeptides
and diverse agonists by the formylpeptide receptors FPR1 and FPR2. Nat. Commun..

[4] Chiang N., Serhan C. N. (2017). Structural elucidation
and physiologic functions of
specialized pro-resolving mediators and their receptors. Mol. Aspects Med..

[5] Dalli J., Winkler J. W., Colas R. A., Arnardottir H., Cheng C.-Y. C., Chiang N., Petasis N. A., Serhan C. N. (2013). Resolvin
D3 and Aspirin-Triggered Resolvin D3 Are Potent Immunoresolvents. Chem. Biol..

[6] Wood M. P., Cole A. L., Eade C. R., Chen L. M., Chai K. X., Cole A. M. (2014). The HIV-1 gp41 ectodomain
is cleaved by matriptase
to produce a chemotactic peptide that acts through FPR2. Immunology.

[7] Serhan C. N. (2014). Pro-resolving
lipid mediators are leads for resolution physiology. Nature.

[8] Bannenberg G. L., Chiang N., Ariel A., Arita M., Tjonahen E., Gotlinger K. H., Hong S., Serhan C. N. (2005). Molecular Circuits
of Resolution: Formation and Actions of Resolvins and Protectins. J. Immunol..

[9] Serhan C. N., Levy B. D. (2018). Resolvins in inflammation:
emergence of the pro-resolving
superfamily of mediators. J. Clin. Invest..

[10] Lucas C. D., Dorward D. A., Tait M. A., Fox S., Marwick J. A., Allen K. C., Robb C. T., Hirani N., Haslett C., Duffin R., Rossi A. G. (2014). Downregulation of Mcl-1 has anti-inflammatory
pro-resolution effects and enhances bacterial clearance from the lung. Mucosal Immunol..

[11] Schmitz
Nunes V., Rogério A. P., Abrahão O. (2020). Insights into
the Activation Mechanism of the ALX/FPR2 Receptor. J. Phys. Chem. Lett..

[12] Chen T., Xiong M., Zong X., Ge Y., Zhang H., Wang M., Won Han G., Yi C., Ma L., Ye R. D., Xu Y., Zhao Q., Wu B. (2020). Structural
basis of ligand binding modes at the human formyl peptide receptor
2. Nat. Commun..

[13] Ge Y., Liao Q., Xu Y., Zhao Q., Wu B., Ye R. D. (2020). Anti-inflammatory
signaling through G protein-coupled receptors. Acta Pharmacol. Sin..

[14] Zhu Y., Lin X., Zong X., Han S., Wang M., Su Y., Ma L., Chu X., Yi C., Zhao Q., Wu B. (2022). Structural
basis of FPR2 in recognition of Aβ42 and neuroprotection by
humanin. Nat. Commun..

[15] Nunes V. S., Abrahão O., Rogério A. P., Serhan C. N. (2023). ALX/FPR2 Activation
by Stereoisomers of D1 Resolvins Elucidating with Molecular Dynamics
Simulation. J. Phys. Chem. B.

[16] Cooray S. N., Gobbetti T., Montero-Melendez T., McArthur S., Thompson D., Clark A. J. L., Flower R. J., Perretti M. (2013). Ligand-specific conformational
change of the G-protein–coupled receptor ALX/FPR2 determines
proresolving functional responses. Proc. Natl.
Acad. Sci. U.S.A..

[17] El
Khamlichi C., Cobret L., Arrang J.-M., Morisset-Lopez S. (2021). BRET Analysis
of GPCR Dimers in Neurons and Non-Neuronal Cells: Evidence for Inactive,
Agonist, and Constitutive Conformations. Int.
J. Mol. Sci..

[18] Jakubík J., El-Fakahany E. E. (2021). Allosteric Modulation of GPCRs of
Class A by Cholesterol. Int. J. Mol. Sci..

[19] Jakubík J., Randáková A. (2022). Insights into the operational model
of agonism of receptor dimers. Expert Opin.
Drug Discovery.

[20] Kasai R. S., Ito S. V., Awane R. M., Fujiwara T. K., Kusumi A. (2018). The Class-A
GPCR Dopamine D2 Receptor Forms Transient Dimers Stabilized by Agonists:
Detection by Single-Molecule Tracking. Cell
Biochem. Biophys..

[21] Nasrallah C., Cannone G., Briot J., Rottier K., Berizzi A. E., Huang C.-Y., Quast R. B., Hoh F., Banères J.-L., Malhaire F., Berto L., Dumazer A., Font-Ingles J., Gómez-Santacana X., Catena J., Kniazeff J., Goudet C., Llebaria A., Pin J.-P., Vinothkumar K. R., Lebon G. (2021). Agonists and allosteric
modulators promote signaling from different
metabotropic glutamate receptor 5 conformations. Cell Rep..

[22] Lomize M. A., Pogozheva I. D., Joo H., Mosberg H. I., Lomize A. L. (2012). OPM database
and PPM web server: resources for positioning of proteins in membranes. Nucleic Acids Res..

[23] Kim S., Thiessen P. A., Bolton E. E., Chen J., Fu G., Gindulyte A., Han L., He J., He S., Shoemaker B. A., Wang J., Yu B., Zhang J., Bryant S. H. (2016). PubChem
Substance and Compound databases. Nucleic Acids
Res..

[24] Schrödinger, L. L. C. Maestro Academic License; Schrödinger Release 2018: New York, NY, 2018.

[25] Vanommeslaeghe K., Hatcher E., Acharya C., Kundu S., Zhong S., Shim J., Darian E., Guvench O., Lopes P., Vorobyov I., Mackerell A. D. (2010). CHARMM general force field: A force
field for drug-like molecules compatible with the CHARMM all-atom
additive biological force fields. J. Comput.
Chem..

[26] Vanommeslaeghe K., MacKerell A. D. (2012). Automation
of the CHARMM general force field (CGenFF)
I: Bond perception and atom typing. J. Chem.
Inf. Model..

[27] Morris G. M., Goodsell D. S., Huey R., Olson A. J. (1996). Distributed automated
docking of flexible ligands to proteins: Parallel applications of
AutoDock 2.4. J. Comput. Aided Mol. Des..

[28] Trott O., Olson A. J. (2010). AutoDock Vina: Improving the speed and accuracy of
docking with a new scoring function, efficient optimization, and multithreading. J. Comput. Chem..

[29] Jo S., Kim T., Iyer V. G., Im W. (2008). CHARMM-GUI: A web-based graphical
user interface for CHARMM. J. Comput. Chem..

[30] Lee J., Cheng X., Swails J. M., Yeom M. S., Eastman P. K., Lemkul J. A., Wei S., Buckner J., Jeong J. C., Qi Y., Jo S., Pande V. S., Case D. A., Brooks C. L., MacKerell A. D., Klauda J. B., Im W. (2016). CHARMM-GUI Input Generator
for NAMD, GROMACS, AMBER, OpenMM, and CHARMM/OpenMM Simulations Using
the CHARMM36 Additive Force Field. J. Chem.
Theory Comput..

[31] Uberuaga B. P., Anghel M., Voter A. F. (2004). Synchronization
of trajectories in
canonical molecular-dynamics simulations: Observation, explanation,
and exploitation. J. Chem. Phys..

[32] Åqvist J., Wennerström P., Nervall M., Bjelic S., Brandsdal B. O. (2004). Molecular
dynamics simulations of water and biomolecules with a Monte Carlo
constant pressure algorithm. Chem. Phys. Lett..

[33] Ryckaert J.-P., Ciccotti G., Berendsen H. J. (1977). Numerical
integration of the cartesian
equations of motion of a system with constraints: molecular dynamics
of n-alkanes. J. Comput. Phys..

[34] Essmann U., Perera L., Berkowitz M. L., Darden T., Lee H., Pedersen L. G. (1995). A smooth particle
mesh Ewald method. J. Chem. Phys..

[35] Brooks B. R., Brooks C. L., Mackerell A. D., Nilsson L., Petrella R. J., Roux B., Won Y., Archontis G., Bartels C., Boresch S., Caflisch A., Caves L., Cui Q., Dinner A. R., Feig M., Fischer S., Gao J., Hodoscek M., Im W., Kuczera K., Lazaridis T., Ma J., Ovchinnikov V., Paci E., Pastor R. W., Post C. B., Pu J. Z., Schaefer M., Tidor B., Venable R. M., Woodcock H. L., Wu X., Yang W., York D. M., Karplus M. (2009). CHARMM: The biomolecular simulation program. J. Comput. Chem..

[36] Huang J., Mackerell A. D. (2013). CHARMM36
all-atom additive protein force field: Validation
based on comparison to NMR data. J. Comput.
Chem..

[37] Case, D. A. ; Ben-Shalom, I. Y. , Brozell, S. R. ; Cerutti, D. S. ; Cheatham, T. E., III ; Cruzeiro, V. W. D. ; Darden, T. A. , Duke, R. E. ; Ghoreishi, D. ; Gilson, M. K. ; Gohlke, H. ; Goetz, A. W. ; Greene, D. ; Harris, R. ; Homeyer, N. ; Huang, Y. ; Izadi, S. ; Kovalenko, A. ; Kurtzman, T. ; Lee, T. S. ; LeGrand, S. ; Li, P. ; Lin, C. ; Liu, J. ; Luchko, T. ; Luo, R. ; Mermelstein, D. J. , Merz, K. M. ; Miao, Y. ; Monard, G. ; Nguyen, C. ; Nguyen, H. ; Omelyan, I. ; Onufriev, A. ; Pan, F. ; Qi, R. ; Roe, D. R. ; Roitberg, A. ; Sagui, C. ; Schott-Verdugo, S. ; Shen, J. ; Simmerling, C. L. ; Smith, J. ; Salomon-Ferrer, J. ; Swails, J. ; Walker, R. C. ; Wang, J. ; Wei, H. ; Wolf, R. M. ; Wu, X. ; Xiao, L. ; York, D. M. ; Kollman, P. A. AMBER 2018. 2018, https://ambermd.org/doc12/Amber18.pdf (accessed May 27, 2025).

[38] Humphrey W., Dalke A., Schulten K. (1996). VMD: Visual
molecular dynamics. J. Mol. Graph..

[39] Miller B. R., McGee T. D., Swails J. M., Homeyer N., Gohlke H., Roitberg A. E. (2012). MMPBSA.py: An Efficient Program for
End-State Free
Energy Calculations. J. Chem. Theory Comput..

[40] Wang E., Sun H., Wang J., Wang Z., Liu H., Zhang J. Z. H., Hou T. (2019). End-Point Binding Free Energy Calculation with MM/PBSA
and MM/GBSA: Strategies and Applications in Drug Design. Chem. Rev..

[41] Genheden S., Ryde U. (2015). The MM/PBSA and MM/GBSA
methods to estimate ligand-binding affinities. Expert Opin. Drug Discovery.

[42] Tan C., Tan Y.-H., Luo R. (2007). Implicit Nonpolar
Solvent Models. J. Phys. Chem. B.

[43] Weiser J., Shenkin P. S., Still W. C. (1999). Approximate atomic surfaces from
linear combinations of pairwise overlaps (LCPO). J. Comput. Chem..

[44] Krishnamoorthy S., Recchiuti A., Chiang N., Yacoubian S., Lee C.-H., Yang R., Petasis N. A., Serhan C. N. (2010). Resolvin
D1 binds human phagocytes with evidence for proresolving receptors. Proc. Natl. Acad. Sci. U.S.A..

[45] Krishnamoorthy S., Recchiuti A., Chiang N., Fredman G., Serhan C. N. (2012). Resolvin
D1 Receptor Stereoselectivity and Regulation of Inflammation and Proresolving
MicroRNAs. Am. J. Pathol..

[46] Miettinen H. M., Mills J. S., Gripentrog J. M., Dratz E. A., Granger B. L., Jesaitis A. J. (1997). The ligand binding
site of the formyl peptide receptor
maps in the transmembrane region. J. Immunol..

[47] Wang L., Yao D., Fan H., Liu H., Xiao Q., Gong W., Wei Z., Zhang C. (2018). Structures
of the Human PGD2 Receptor CRTH2 Reveal
Novel Mechanisms for Ligand Recognition. Mol.
Cell.

[48] Rogerio A. P., Haworth O., Croze R., Oh S. F., Uddin M., Carlo T., Pfeffer M. A., Priluck R., Serhan C. N., Levy B. D. (2012). Resolvin D1 and
Aspirin-Triggered Resolvin D1 Promote
Resolution of Allergic Airways Responses. J.
Immunol..

[49] Yu Y., Ye R. D. (2015). Microglial Aβ
Receptors in Alzheimer’s Disease. Cell.
Mol. Neurobiol..

[50] Ansari J., Kaur G., Gavins F. N. E. (2018). Therapeutic
potential of annexin
A1 in ischemia reperfusion injury. Int. J. Mol.
Sci..

[51] Corminboeuf O., Leroy X. (2015). FPR2/ALXR agonists
and the resolution of inflammation. J. Med.
Chem..

[52] Fiore S., Romano M., Reardon E. M., Serhan C. N. (1993). Induction of functional
lipoxin A4 receptors in HL-60 cells. Blood.

[53] Fiore S., Maddox J. F., Perez H. D., Serhan C. N. (1994). Identification of
a human cDNA encoding a functional high affinity lipoxin A4 receptor. J. Exp. Med..

[54] Zhang S., Gong H., Ge Y., Ye R. D. (2020). Biased allosteric
modulation of formyl peptide receptor 2 leads to distinct receptor
conformational states for pro- and anti-inflammatory signaling. Pharmacol. Res..

[55] Ballante F., Kooistra A. J., Kampen S., de Graaf C., Carlsson J. (2021). Structure-Based
Virtual Screening for Ligands of G Protein–Coupled Receptors:
What Can Molecular Docking Do for You?. Pharmacol.
Rev..

[56] Bjarnadóttir T.
K., Gloriam D. E., Hellstrand S. H., Kristiansson H., Fredriksson R., Schiöth H. B. (2006). Comprehensive repertoire and phylogenetic
analysis of the G protein-coupled receptors in human and mouse. Genomics.

[57] Joost P., Methner A. (2002). Phylogenetic analysis
of 277 human G-protein-coupled
receptors as a tool for the prediction of orphan receptor ligands. Genome Biol..

[58] Katritch V., Cherezov V., Stevens R. C. (2012). Diversity
and modularity of G protein-coupled
receptor structures. Trends Pharmacol. Sci..

[59] Chávez-Castillo M., Ortega A. ´., Cudris-Torres L., Duran P., Rojas M., Manzano A., Garrido B., Salazar J., Silva A., Rojas-Gomez D. M., De Sanctis J. B., Bermúdez V. (2021). Specialized
Pro-Resolving Lipid Mediators: The Future of Chronic Pain Therapy?. Int. J. Mol. Sci..

[60] Serhan C. N., Levy B. D. (2025). Proresolving Lipid
Mediators in the Respiratory System. Annu. Rev.
Physiol..

[61] Serhan C. N., Sulciner M. L. (2023). Resolution medicine
in cancer, infection, pain and
inflammation: are we on track to address the next Pandemic?. Cancer Metastasis Rev..

