# Supplementary material for: ALX/FPR2 Receptor Activation by Inflammatory (fMLFII) and Pro-resolving (LXA4 and RvD3) Agonists
Source: ACS Phys Chem Au. 2025 Jun 6;5(4):367–74. doi: 10.1021/acsphyschemau.5c00008 (PMC12291131; doi:10.1021/acsphyschemau.5c00008)
Supplement: Supplementary file 1 [file pg5c00008_si_001.pdf]

Supporting Information

ALX/FPR2 receptor activation by inflammatory (fMLFII) and pro-resolving (LXA_4 and RvD3) agonists

Vinicius S. Nunes[1,2 &], Charles N. Serhan[3], Odonírio Abrahão Jr.[4], and Alexandre P. Rogério[5]

1 – Programa de Pós-Graduação em Produtos Bioativos e Biociências, Universidade Federal do Rio de Janeiro, Macaé, Rio de Janeiro, Brasil.

2 – Laboratório Nacional de Computação Científica, Petrópolis, Rio de Janeiro, Brasil.

3 – Center for Experimental Therapeutics and Reperfusion Injury, Department of Anesthesiology, Perioperative and Pain Medicine, MassGeneral Brigham (MGB) and Harvard Medical School, Boston, Massachusetts, USA.

4 – Laboratório de Química Computacional Medicinal, Universidade Federal do Triângulo Mineiro, Uberaba, Minas Gerais, Brasil.

5 – Laboratório de Imunofarmacologia Experimental, Universidade Federal do Triângulo Mineiro, Uberaba, Minas Gerais, Brasil.

[&]Corresponding Author: viniciusschmitz@macae.ufrj.br

Additional experimental details, figures and tables that complement the results obtained in the present work.

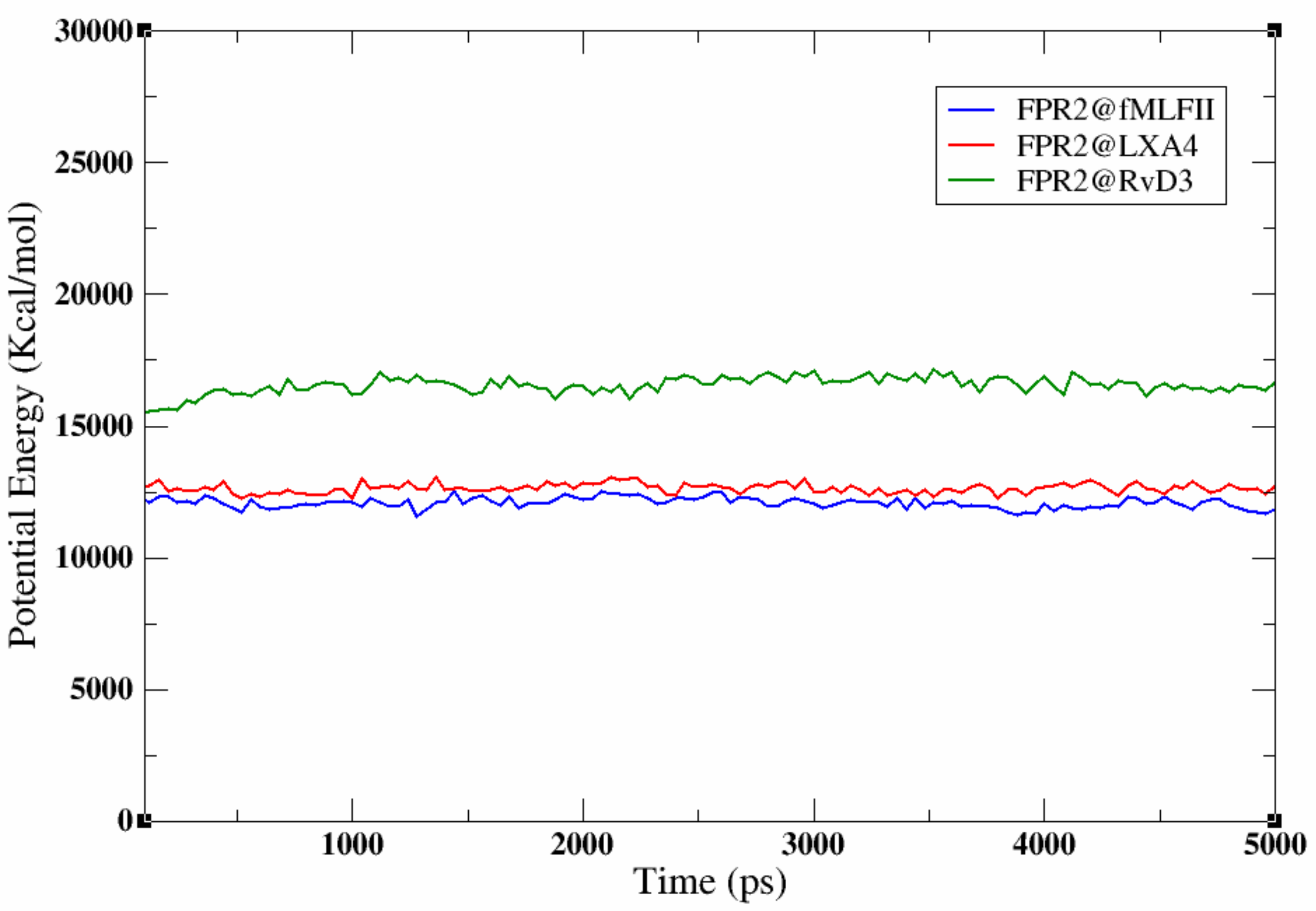

Figure S1: Total potential energy (PE) of the membrane and protein in the last 5000ps of the equilibration. PE BLT1@fMLFII system (blue line), PE BLT1@LXA$_4$ system (red line), PE BLT1@RvD3 system (green line).

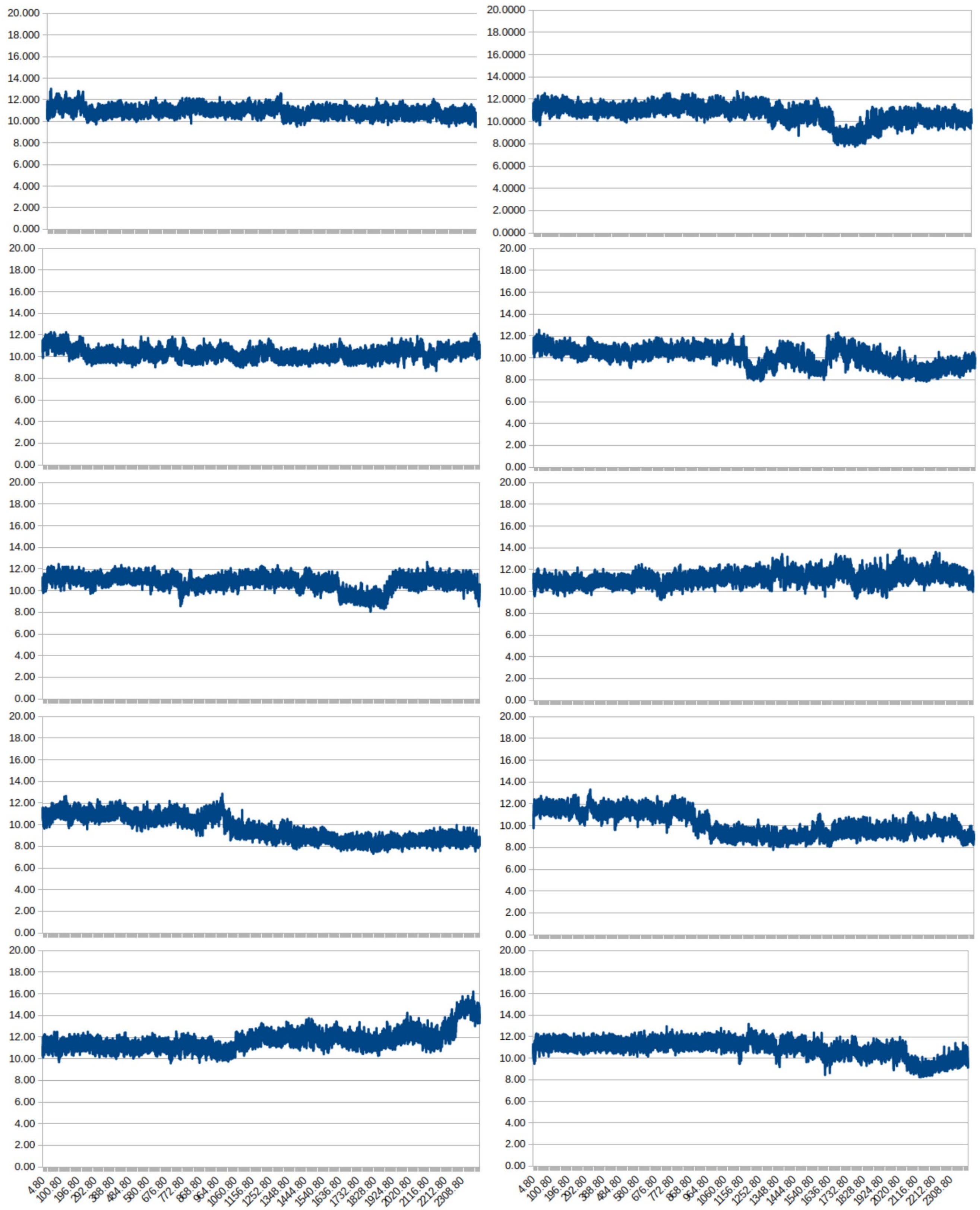

Figure S2: Average TMH3-TMH6 distance variation on each FPR2@fMLFII simulations.

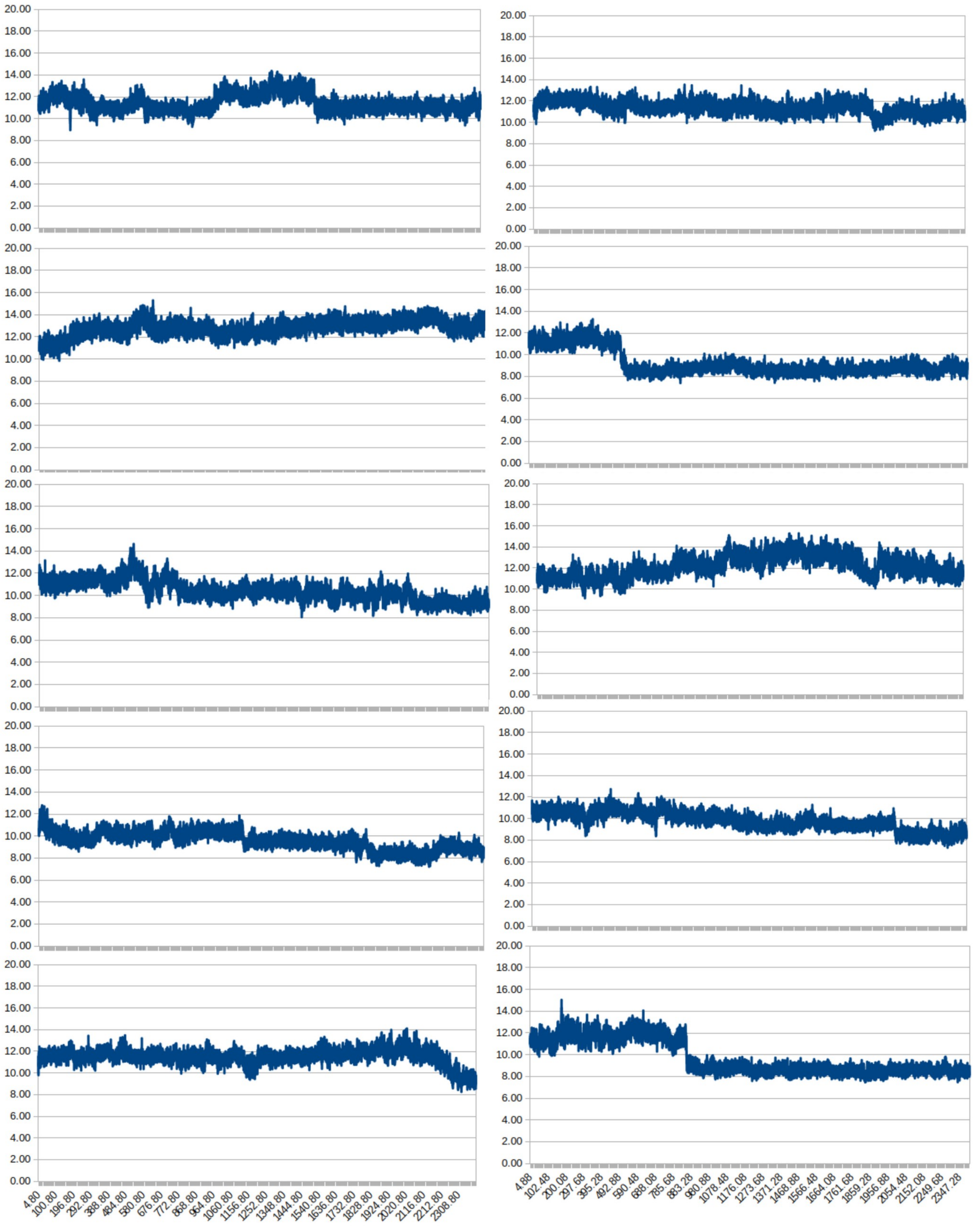

Figure S3: Average TMH3-TMH6 distance variation on each FPR2@LXA_4 simulations.

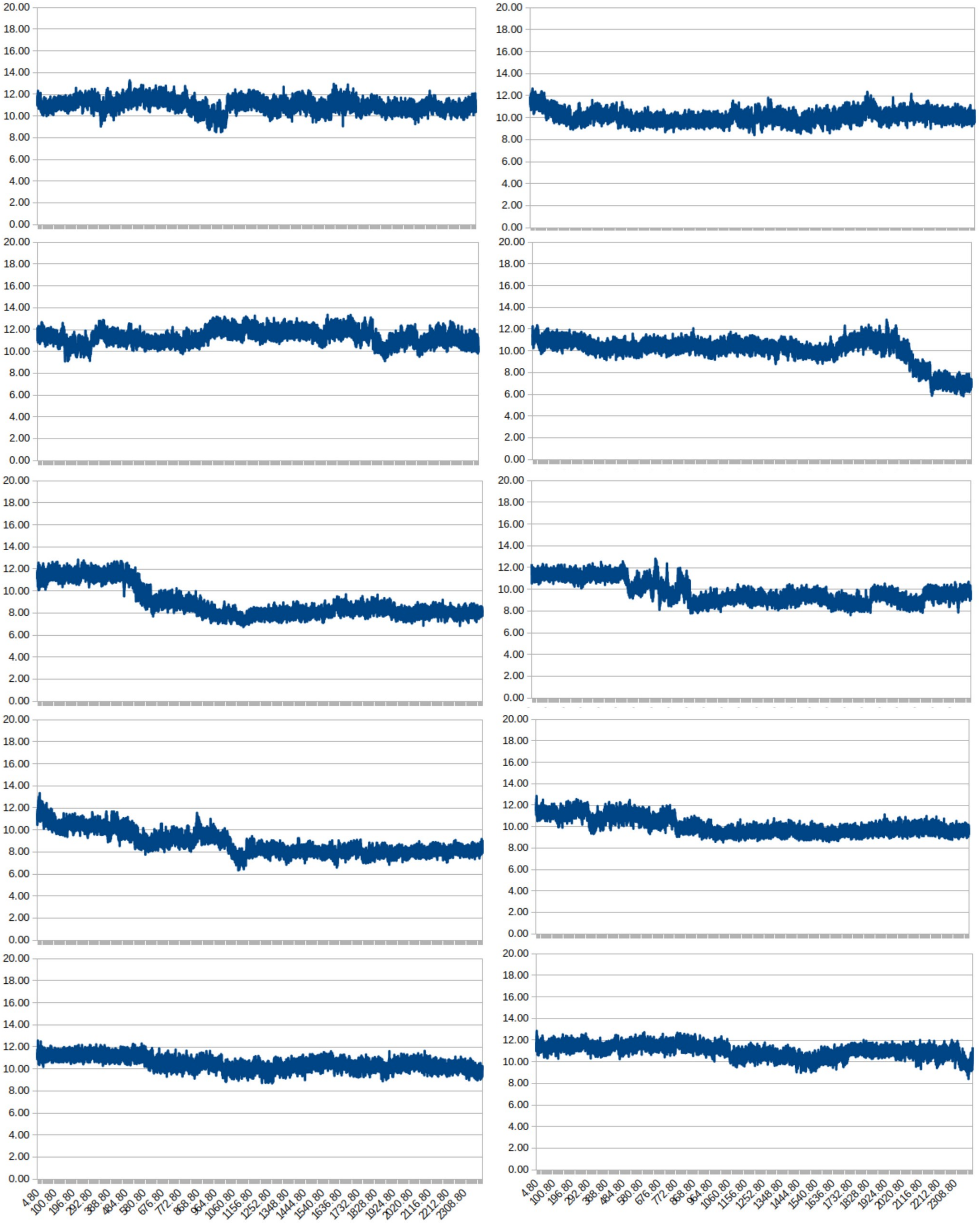

Figure S4: Average TMH3-TMH6 distance variation on each FPR2@RvD3 simulations.

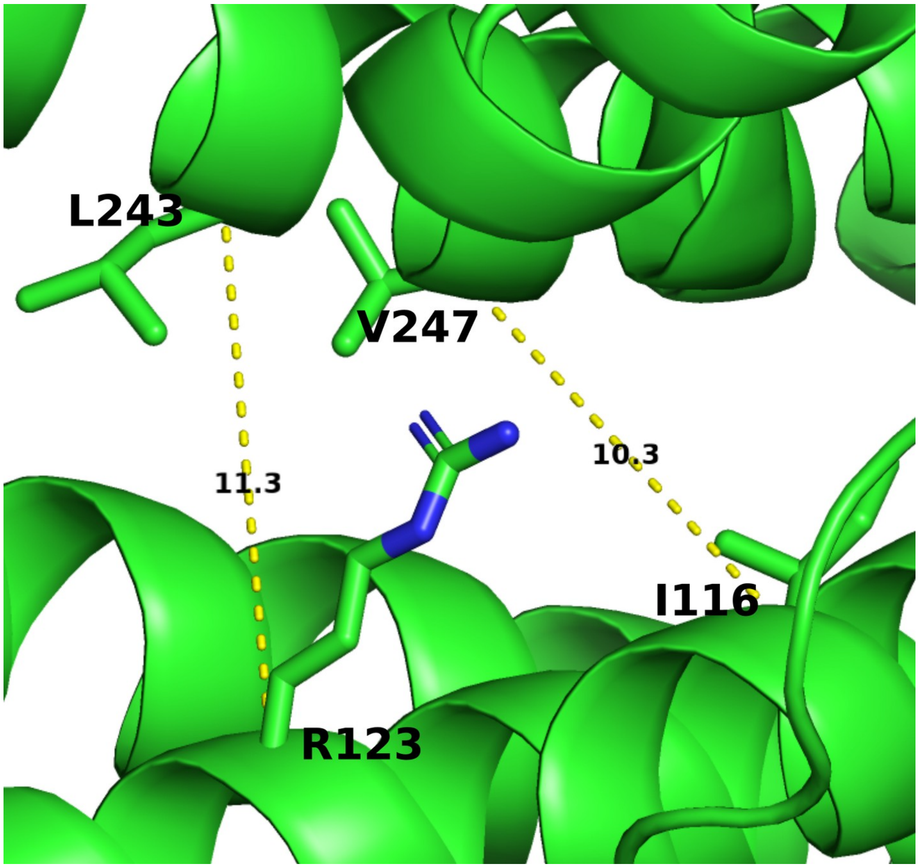

Figure S5: I116-V247 and R123-L243 Cα distances.

	S1	S2	S3	S4	S5	S6	S7	S8	S9	S10	Avg.	SD
**FPR2@fMLFII**	0.23	0.65	0.33	0.9	0.52	0.21	0.57	0.79	0.15	0.6	0.5	0.26
**FPR2@LXA4**	0.11	0.8	0.32	0.85	0.92	0.18	0.89	0.14	0.99	0.35	0.56	0.36
**FPR2@RvD3**	0.42	0.65	0.21	0.17	0.29	0.78	0.07	0.22	0.43	0.67	0.39	0.24

Table S1: Frames frequency where average distance TMH3-TMH6 were >= 10.8Å, for all simulations.

	S1	S2	S3	S4	S5	S6	S7	S8	S9	S10	Avg.	SD
**K90**	0.00	0.36	0.00	0.00	0.00	0.00	0.00	0.60	0.31	0.21	0.15	0.21
**D106**	0.94	0.88	0.97	0.96	0.97	0.99	0.97	0.91	0.07	0.98	**0.87**	**0.28**
**T177**	0.25	0.36	0.00	0.00	0.26	0.20	0.29	0.52	0.73	0.35	0.30	0.22
**F178**	0.00	0.00	0.00	0.00	0.00	0.00	0.00	0.00	0.61	0.00	0.06	0.19
**R201**	0.94	0.44	0.37	0.80	0.39	0.68	0.40	0.50	0.28	0.40	**0.52**	**0.21**
**R205**	0.11	0.62	0.53	0.93	0.52	0.24	0.70	0.91	0.17	0.64	**0.54**	**0.29**
**Q258**	0.00	0.00	0.00	0.52	0.00	0.21	0.00	0.00	0.00	0.00	0.07	0.17
**K278**	0.22	0.00	0.00	0.00	0.00	0.00	0.00	0.00	0.00	0.00	0.02	0.07

Table S2: Non-covalent interactions frequency in the FPR2@fMLFII simulations. Frequency >= 20%.

	S1	S2	S3	S4	S5	S6	S7	S8	S9	S10	Avg.	SD
**S84**	0.00	0.00	0.00	0.22	0.00	0.00	0.37	0.00	0.00	0.00	0.06	0.13
**C176**	0.00	0.00	0.30	0.00	0.00	0.00	0.00	0.25	0.00	0.00	0.05	0.12
**T177**	0.00	0.00	0.33	0.00	0.00	0.27	0.00	0.00	0.00	0.00	0.06	0.13
**R201**	0.41	0.63	0.42	0.92	0.70	0.29	0.72	0.41	0.86	0.94	**0.63**	**0.24**
**R205**	0.34	0.87	0.41	0.95	0.96	0.25	0.93	0.31	0.98	0.35	**0.64**	**0.32**
**T265**	0.00	0.00	0.00	0.00	0.23	0.00	0.00	0.00	0.00	0.00	0.02	0.07
**K276**	0.37	0.25	0.38	0.00	0.00	0.30	0.00	0.00	0.00	0.00	0.13	0.17

Table S3: Non-covalent interactions frequency in the FPR2@LXA 4 simulations. Frequency >= 20%.

	S1	S2	S3	S4	S5	S6	S7	S8	S9	S10	Avg.	SD
**S84**	0.00	0.00	0.00	0.00	0.40	0.00	0.00	0.00	0.00	0.00	0.04	0.13
**T177**	0.31	0.00	0.00	0.00	0.21	0.00	0.00	0.00	0.00	0.00	0.05	0.11
**R190**	0.00	0.00	0.00	0.00	0.00	0.00	0.37	0.00	0.00	0.00	0.04	0.12
**R201**	0.83	0.80	0.27	0.70	0.40	0.80	0.01	0.57	0.85	0.70	**0.59**	0.28
**R205**	0.44	0.64	0.33	0.18	0.44	0.88	0.08	0.17	0.51	0.84	**0.45**	0.27
**N285**	0.00	0.00	0.23	0.00	0.00	0.00	0.00	0.00	0.00	0.00	0.02	0.07

Table S4: Non-covalent interactions frequency in the FPR2@RvD3 simulations. Frequency >= 20%.

ΔGBind	S1	S2	S3	S4	S5	S6	S7	S8	S9	S10	Avg	SD
**fMLFII**	-10.76	-12.32	-12.09	-13.96	-9.68	-12.55	-12.87	-12.54	-2.75	-10.88	-11.04	3.16
**LXA4**	-5.61	-11.60	-6.28	-13.14	-10.89	-5.09	-11.10	-3.09	-12.50	-9.38	-8.87	3.55
**RvD3**	-5.82	-7.39	-2.92	-3.08	-5.46	-11.81	-2.01	-5.61	-8.36	-11.11	-6.36	3.34

Table S5: Average of MMPBSA calculations for three complexes on each simulation (energy in kcal/mol).

ΔGBind	S1	M_E	S2	M_E	S3	M_E	S4	M_E	S5	M_E	S6	M_E	S7	M_E	S8	M_E	S9	M_E	S10	M_E
**fMLFII**	-10.76	0.16	-12.32	0.37	-12.09	0.58	-13.96	0.94	-9.68	0.99	-12.55	0.86	-12.87	0.81	-12.54	0.26	-2.75	1.19	-10.88	0.46
**LXA4**	-5.61	0.35	-11.6	0.11	-6.28	0.14	-13.14	0.48	-10.89	0.24	-5.09	0.76	-11.1	0.26	-3.09	0.84	-12.5	0.37	-9.38	0.15
**RvD3**	-5.82	0.66	-7.39	0.43	-2.92	0.81	-3.08	0.97	-5.46	0.74	-11.81	0.49	-2.01	0.76	-5.61	0.70	-8.36	0.14	-11.11	0.55

Table S6: Average of MMPBSA calculations for three complexes on each simulation, and error of average (M_E). Energy in kcal/mol.

	D106	R201	R205
**fMLFII**	-2.27	-21.02	-29.61
**LXA4**	1.43	-37.20	-39.30
**RvD3**	1.37	-33.56	-18.76

Table S7: Per-residue decomposition for the three complexes. The average for 10 simulations in kcal/mol.

Lig	EC50	ΔG_Bind=RTln(EC50) (kcal/mol)	Ref.	Avg. PBSA (kcal/mol)	S.D. PBSA (kcal/mol)
LXA4	8.30E-10	-12.39	PMID: 9054386	-8.87	±3.55
RvD3	1.00E-07	-9.55	PMID:27534559	-6.36	±3.64
fMLFII	3.80E-09	-11.49	PMID:24285541	-11.04	±3.16

Table S8: MMPBSA calculations versus experimental ΔG_{Bind}.
